# Novel AIDS vaccine approach using epithelial stem cells as mucosal antigen-presenting cells

**DOI:** 10.1186/1742-4690-9-S2-P355

**Published:** 2012-09-13

**Authors:** G Bonello, N Chenciner, R White, M Salas, P Blancou, M Gauduin

**Affiliations:** 1 Texas Biomedical Research Institute, San Antonio, TX, USA; 2 Institut Pasteur, Paris, France; 3 Universitée de Nantes, Nantes, France

## Background

Because HIV transmission occurs predominantly across mucosal surfaces, the ideal vaccine strategy to prevent infection would be to target HIV at mucosal entry sites of transmission. We investigated a novel vaccine approach, which aim is to elicit long-term immunity against HIV infection at the entry site of the virus. This strategy relies on the expression of viral proteins from epithelial stem cells at the basal layer of the epithelium and using a promoter that is specific for terminally differentiated epithelial.

## Methods

The involucrin promoter, which is exclusively expressed in terminally differentiated epithelial cells, was chosen and used as a tool. We generated a GFP-tagged replication competent SIVdeltaNef and a GFP-tagged replication deficient SIVdeltaVifdeltaNef constructs under the transcriptional control of the involucrin promoter (pINV). Viral stocks used to deliver these constructs to basal epithelial cells were obtained by their co-transfection with a plasmid encoding for VSV-G envelope proteins used as pseudotyping envelope protein of significantly broadened host cell range.

## Results

When administered intradermally to mice, we found that GFP-reporter gene under the transcriptional control of the involucrin promoter was expressed in the upper layers of the epidermis. Although transduced cells were very low in number, high and sustained anti-GFP antibody production was observed in vivo. After production of high concentrations of infectious viral particles, we demonstrated the integrity of our constructs (regions encoding for GAG, POL and GFP) in the VSV-G pseudotyped viral particles to be used for inoculation in nonhuman primate model for AIDS.

## Conclusion

After integration of pINV-driven constructs, basal layer cells will divide and differentiate thus triggering SIV antigens expression as well as both direct and cross priming. Long-term antigen expression in upper layers of the epithelium may occur even after multiple cycles of epithelia renewal, thus eliciting a long-term immunity against HIV/SIV infection at the site.

